# 2066: Television viewing: Associations with eating behavior and cravings in healthy, non-obese young adults

**DOI:** 10.1017/cts.2017.144

**Published:** 2018-05-10

**Authors:** Amanda E. Staiano, Corby K. Martin, Jennifer C. Rood, Peter T. Katzmarzyk

## Abstract

OBJECTIVES/SPECIFIC AIMS: The majority of obese adults do not become obese until adulthood. Although adults spend the equivalent of a 40-hour work week in front of the television (TV), there are mixed data on whether the sedentary behavior of TV viewing is linked with weight gain during adulthood. The purpose of this study was to examine the associations among sedentary behavior, measured as TV viewing and TV in the bedroom, with eating behavior, eating attitudes and cravings, fat gain, and blood pressure in healthy young adults over a 2-year period. METHODS/STUDY POPULATION: The sample included 73 healthy, nonobese adults (56% women, 80% white) who were 26.8±4.5 years of age with a body mass index of 22.9±2.4 kg/m^2^. Participants completed clinic visits at baseline and 2-years later (Year 2) which assessed weight, height, blood pressure, waist circumference, and total body fat measured by dual energy X-ray absorptiometry. A food frequency questionnaire was used to estimate dietary intake, and the eating inventory was used to assess dietary restraint, disinhibition, and hunger. At baseline, participants self-reported TV habits including number of hours/week of watching TV (including cable, VCR, DVD) and presence of a TV in the bedroom. For the analysis, participants were stratified by quartiles of TV viewing time. *T* tests were used to examine the association between TV viewing and bedroom TV. Linear regression models were used to examine the association between TV viewing and each anthropometric and body composition measure and change over the 2-year period, as well as with the dietary constructs. Models controlled for age, sex, and baseline body fat. Separate models were used to investigate the associations between bedroom TV and the same dependent variables. RESULTS/ANTICIPATED RESULTS: Participants reported an average of 13.3±10.8 hours/week of TV viewing, with 33.3% reporting a TV in the bedroom. There were no differences in age, sex, or race among the quartiles of TV viewing or between those who did and did not have a bedroom TV. Adults with a bedroom TV did not differ in hours/week of TV viewing compared with those without a bedroom TV. Amount of TV viewing was associated with higher systolic blood pressure at baseline (*p*=0.05) but with no other anthropometric or body composition indices nor with change in body composition over the 2-year period. Adults with a bedroom TV reported higher craving for sweets at baseline (*p*=0.03). Amount of TV viewing was related to lower consumption of vegetables (*p*=0.04) and fruit or fruit juice (*p*=0.03) at Year 2, but there was no association with total calorie consumption. TV viewing and bedroom TV were not related to dietary restraint, disinhibition, or hunger at either time point. DISCUSSION/SIGNIFICANCE OF IMPACT: Adults who watched more TV consumed fewer fruits and vegetables, and those with a TV in the bedroom reported higher craving for sweets. Though there were no observed relationships between TV habits and body composition change, the associations with cravings and food consumption warrant further exploration. Querying young adults’ TV and media use habits in clinical settings may alert physicians to those at risk of developing poor dietary habits.

